# Evaluation of the relationship between insulin resistance and visceral adiposity index in patients with acne vulgaris

**DOI:** 10.3906/sag-2011-304

**Published:** 2021-12-21

**Authors:** İbrahim Fuat Kayıhan KAYA, Mehmet Ali ERYILMAZ, Selma PEKGÖR, Orhan KÜLAHCI

**Affiliations:** 1 Family Medicine, Derebucak Family Health Center, Konya, Turkey; 2 Department of General Surgery and Acting-Director of Family Medicine Clinic, Konya Health Application and Research Center, University of Health Sciences, Konya, Turkey; 3 Department of Family Medicine, Konya Health Application and Research Center, University of Health Sciences, Konya, Turkey; 4 Department of Dermatology, Medicana Konya Hospital, Karatay University, Konya, Turkey

**Keywords:** Acne vulgaris, insulin resistance, visceral adiposity index

## Abstract

**Background/aim:**

Acne vulgaris (AV) is a chronic inflammatory skin disease that is common among adolescents. Its etiology involves hormones, diet, genetics, and environmental factors. The aim of this study was to investigate the association between acne vulgaris and insulin resistance (IR) and visceral adiposity index (VAI).

**Materials and methods:**

The study included 184 individuals. Participants’ height, weight, waist and hip circumference and blood pressure were measured. Morning fasting blood was obtained from all participants for measuring glucose, insulin, triglyceride (TG), and cholesterol levels. The HOMA index and visceral adiposity index were calculated. The collected data were analyzed using statistical methods.

**Results:**

The patient and control groups exhibited similar age, gender, and body mass index (p > 0.05). There was no significant difference between the patient and control groups in HOMA index and VAI (p > 0.05). A positive correlation was observed between VAI and glucose, insulin, HOMA index, TG level, and waist circumference, whereas there was a negative correlation with high-density lipoprotein (HDL).

**Conclusion:**

Our study results demonstrated that IR and VAI were similar both in the AV and healthy control groups. There was no correlation between acne severity score and waist circumference, glucose, insulin, TG, HDL, HOMA index, and VAI. These results indicate that IR and visceral adipose tissue dysfunction alone are not effective in the development of acne.

## 1. Introduction

Acne vulgaris is a common skin disease in adolescence, varies by age, gender, ethnicity, and different geographical regions. Its incidence is 64% in 20s, 43% in 30s and 3%–5% in 40s. Approximately 20% of individuals with acne suffer from the moderate and severe form of acne [1] . There are four main mechanisms involved in the pathogenesis of AV: follicular hyperkeratinization, increased sebum production, *Propionibacterium acnes* colonization, and inflammation. These mechanisms are believed to give rise to the disease with the interaction of genetic susceptibility, diet, environmental factors, hormonal and immune systems [2]. Androgens, growth hormones, and various growth factors play a role in sebaceous gland growth and differentiation. Insulin and insulin-like growth factor-1 receptors reside on the surface of keratinocytes, which are involved in the growth and differentiation of sebocytes and keratinocytes [3]. Proinflammatory cytokines emerging under conditions of chronic inflammation also cause IR and increase the secretion of insulin by the pancreas. Elevated insulin levels in adolescence lead to increased serum androgen levels and stimulate sebum and keratinocytes in the skin, leading to the development of acne lesions [3,4]. Previous studies have shown that IR is associated with a higher incidence of skin diseases such as acne, psoriasis, alopecia areata, and vitiligo [4]. The VAI is a recently developed method that accurately shows the amount of visceral adipose tissue. The VAI, which is calculated using anthropometric measures (waist circumference, body mass index (BMI)) and lipid levels (TG, HDL), is an easy mathematical index with high sensitivity and specificity in demonstrating gender-specific visceral adipose tissue dysfunction [5].

The aim of this study was to investigate the association between acne vulgaris, a chronic inflammatory skin disease, and VAI, an indicator of IR and visceral adipose tissue dysfunction.

## 2. Materials and methods

The study was designed as a prospective cross-sectional study evaluating the association between AV and IR and VAI. The ethics committee approval was obtained from Selçuk University Faculty of Medicine (2018/07). A total of 92 patients diagnosed with moderate to severe acne between 18–35 years of age who were admitted to the dermatology outpatient clinic between January 2018 and December 2018 were included in the study. A total of 92 healthy individuals without AV who were admitted to the family practice center and dermatology outpatient clinic were included in the control group. Acne vulgaris and control group equalized in terms of age and BMI. All participants were informed about the study in accordance with the Declaration of Helsinki, and those who agreed were included in the study with their signed consents being obtained. Diabetes, hypertension, thyroid disease, liver disease, endocrinological diseases (cushing syndrome, acromegaly, congenital adrenal hyperplasia, polycystic ovary syndrome (anamnesis and pelvic usg), etc), smokers, and those who used isotretinoin, steroids, and hormonal contraceptives were excluded from the study. Similarly, since diabetes drugs can affect insulin resistance, those who use diabetes drugs were not included in the study. Participants’ height, weight, waist and hip circumference and blood pressure were measured. BMI was calculated by dividing the patient’s weight in kg by the square of their height in meters. The Global Acne Grading System developed by Doshi et al. was used to determine the severity of acne [6].

The Global Acne Grading System divides seborrheic areas into 6 parts: the right cheek, left cheek, nose, chin, and chest and upper back. Each region is given a value depending on severity (forehead = 2, right cheek = 2 left cheek = 2, nose = 1, chin = 1, upper back and chest = 3) and the local score is calculated by multiplying this value by the lesion grade (no lesions = 0, comedones = 1, papules = 2, pustules = 3, nodules = 4). Total score ranges from 0 (no acne), 1–18 (mild acne), 19–30 (moderate acne), 31–38 (severe acne), and 39–44 (very severe acne).

Venous blood specimens were taken into the collection tubes in the morning following 12 h of fasting for biochemical and hormonal analysis. Blood specimens were analyzed on the same day at the biochemistry laboratory of hospital. Glucose was analyzed with the enzymatic method on Beckman Coulter AU 5800 (Beckman Coulter, Inc., CA 92821, USA). Total cholesterol, HDL cholesterol and TG were analyzed with the enzymatic/colorimetric method on Beckman Coulter AU 5800 (Beckman Coulter, Inc., CA 92821, USA). Insulin was analyzed with the direct chemiluminescence method on Siemens Immulite 2000 (USA). IR was determined using the formula of the HOMA index = (fasting glucose (mg/dL) × fasting insulin (mIU/L)) ÷ 405. The HOMA index above 2.5 was considered IR [7]. The VAI was calculated using the following gender-specific formulas.

For men; VAI = [Waist circumference ÷ (39.68 + (1.88 × BMI))] × (TG ÷ 1.03) × (1.31 ÷ HDL-cholesterol).

For women; VAI = [Waist circumference ÷ (36.58 + (1.89 × BMI))] × (TG ÷ 0.81) × (1.52 ÷ HDL-cholesterol).

Waist circumference: cm; TG ve HDL-C: mmol/l.

The collected data were compared in the patient and control groups. Additionally, multiple group comparisons were performed between moderate and severe acne and control groups.

Power analysis was performed using the GPower 3.1 software package. As a result of the power analysis, the total number of observations required for 5% significance, 0.5 effect size and 85% power was determined as 146. Our study was carried out with a total of 184 individuals. All statistical analyses were performed using the Statistical Package for Social Sciences for Windows version 22.0 (SPSS). The Kolmogorow-Smirnow and Shapiro-Wilk tests were used to test whether the data conformed to normal distribution. The student-t test was used for the normally distributed data in the two group comparisons while the Mann-Whitney U test was used for the nonnormally distributed data. Multiple comparisons of parametric data one-way analysis of variance (ANOVA) test was used. Kruskal-wallis test was used to compare multiple groups that did not show normal distribution. The chi-square test was used for comparing the categorical variables. Multiple comparison tests were not performed for nonsignificant values as a result of ANOVA and Kruskal-Wallis tests. The Pearson correlation was used to determine the relationship between normally distributed data, whereas the Spearman correlation analysis was used for nonnormally distributed data. The data with normal distribution are presented as mean standard error, whereas the data without normal distribution are presented as median (25–75 quarter), and categorical data as frequency. The value p < 0.05 was considered statistically significant.

## 3. Results

The study was completed with a total of 92 AV patients and 92 healthy controls. The patients and controls exhibited similar age, gender, and BMI (p > 0.05). There were no obese participants in the patient or control group. Demographic characteristics of the participants are shown in Table1.

The HOMA scores exhibited similar results, with 1.749 (1.005–3.573) in the patient group and 1.78 (1.040–3.038) in the control group (p = 0.704). There were 30 individuals with IR in both the patient and control groups (p = 1.000). The evaluation of the VAI scores revealed no significant difference between the two groups, with the calculated VAI score of 1.123 (0.792–1.538) in the patient group, and 1.075 (0.803–1.45) in the control group (p = 0.879) (Table 2).

Patients with mild acne were excluded from the study. In the patient group, 50 (54%) individuals had moderate and 42 (46%) had severe acne. The three group comparisons of the control, moderate, and severe acne groups demonstrated no significant difference in IR (p = 0.590) and VAI (p = 0.947). Other parameters were similar in all three groups. The VAI score was higher in those with severe AV, but no statistically significant difference was found (Table 3).

The correlation analysis of metabolic and anthropometric measures revealed a positive correlation between the VAI score and waist circumference, hip circumference, HOMA index, insulin, glucose and TG, and a negative correlation with HDL. A positive correlation was observed between the HOMA index and BMI, insulin, glucose and TG (Table 4). There was no correlation between acne severity score and study parameters in the patient group (Table 5).

## 4. Discussion

In the present study, a total of 92 individuals with moderate to severe acne vulgaris and 92 healthy individuals in the control group were compared in terms of IR and VAI. Our literature review demonstrated that no study has been conducted on VAI to date, which is accepted as an indicator of cardiometabolic risk in patients with AV. This is the first study in which IR and VAI were evaluated together in the acne vulgaris and control groups. Our study results demonstrated similar HOMA-IR and VAI levels between patients with acne vulgaris and the healthy control group. A positive correlation was found between VAI and glucose, insulin, HOMA-IR, hip circumference.

Hormones play important role in the pathogenesis of AV. Androgens, growth hormone, insulin and IGF-1 levels increase in puberty [8]. Insulin and IGF-1 are known to increase acne formation by increasing sebum production and conversion of testosterone to dihydrotestosterone [9]. While some studies reported that individuals with AV had high serum insulin and HOMA values, and there was a weak positive correlation between acne severity score and insulin and HOMA [10,11], other studies reported that patient and control groups had similar serum glucose, insulin and HOMA levels and there was no relationship between acne severity score and insulin and HOMA-IR [12–14]. In our study, we did not find any difference between the patient and control groups in terms of fasting glucose, insulin, and HOMA-IR. We did not find any relationship between acne severity score and fasting glucose, insulin, and HOMA-IR.

It was shown that adipokines and proinflammatory cytokines secreted by visceral adipose tissue are associated with chronic inflammation, IR, type 2 diabetes, hypertension, and atherogenic dyslipidemia. Visceral adipose tissue dysfunction increases the risk of mortality of these diseases, independently of BMI [15]. VAI can be used as a marker of visceral adipose tissue dysfunction, which can be easily calculated using both anthropometric measures and lipid levels [16]. Another study compared waist circumference, BMI, waist-hip ratio, waist-height ratio, VAI, TG/HDL ratios in predicting 10-year cardiovascular disease risk. It was emphasized that VAI is the most sensitive parameter in predicting cardiovascular disease risk. A significant relationship between VAI and diabetes and hypertension were reported [17]. Similarly, another study examining the relationship between VAI with IR reported that the VAI score was significantly higher in overweight and obese individuals with IR [18]. In our study, we found that VAI is related to weight, hip circumference, HOMA-IR, glucose. Ji et al. examined the relationship between VAI and HOMA-IR in adults. They reported a significant positive relationship between the HOMA-IR and VAI following the improvement of waist/height ratio, waist/hip ratio and lipid parameters in individuals with normal waist circumference, with and without diabetes [19]. A positive correlation was found between VAI and insulin and HOMA-IR [18,20]. The results of our study showed that there was a positive correlation between VAI and hip circumference, HOMA-IR, insulin, glucose; and a negative correlation between VAI and HDL.

While past studies in the literature investigated only IR in patients with AV, our study examined IR and VAI, which is a very good marker in predicting visceral adipose tissue dysfunction. The evaluation of the patient and control groups together along with the exclusion of those who were smokers, who had the polycystic ovarian syndrome, metabolic diseases, and those who used drugs that would affect metabolism are among the strengths of our study. The low number of men is the limitation of our study.

Our study results demonstrated that the frequency of IR and VAI was similar in the acne vulgaris and healthy control groups. No relationship was found between acne severity score and waist circumference, glucose, insulin, TG, HDL, HOMA-IR and VAI. These results suggest that IR and visceral adipose tissue dysfunction are not individually effective in the development of AV. Studies involving acne patients with obesity and controls are needed. In this regard, we believe that prospective studies with broad exclusion criteria and a greater number of participants should be conducted.

Presentation at a meeting: Yes, oral presentations.

Organisation: 19th International Eastern Mediterranean Family Medicine Congress, Place: Adana /TURKEY

Date: 17th – 20th September 2020

Source(s) of support: no

Conflicting interest (If present, give more details): no

## Figures and Tables

**Table 1:** Demographic characteristics of the participants.

Characteristics		Control group (n = 92)	Patient group (n = 92)	P-value
Age (year)		21 (19–23)	22 (19–25)	0.157 a
Gender n (%)	Women	80 (87%)	85 (92.4%)	
	Men	12 (13%)	7 (7.6%)	0.226 b
Body mass index (kg/m^2^)		21.50 ± 0.26	21.17 ± 0.22	0.334 c
Income n (%)	Less than 3000 TL	77 (83.7%)	81 (88%)	
	More than 3000 TL	15 (16.3%)	11 (12%)	0.526 b

Parametric data were given as mean–standard error and nonparametric data were presented as median (25–75 quartile) and categorical variables were presented as a percentage.

a Mann Whitney U,

b Chi-square test,

c Student–t test.

**Table 2:** Comparison of data in the patient and control groups.

	Control group (n = 92)	Patient group (n = 92)	P-value
Age (year)	22 (19–25)	21 (19–23)	0.157 a
Height (m)	1.65 ± 0.01	1.64 ± 0.01	0.114 b
Weight (kg)	59 (54–64.5)	56 (52–61.5)	0.092 a
BMI (kg/m^2^)	21.50 ± 0.26	21.17 ± 0.22	0.334 b
Waist circumference (cm)	74 (68.5–79.5)	72 (68–78.5)	0.488 a
Hip circumference (cm)	92.5 (90–97)	93 (89–97)	0.861 ad
Waist/hip ratio	0.77 (0.74–0.83)	0.77 (0.75–0.81)	0.822 ad
Systolic blood pressure (mmhg)	110 (100–112.5)	105 (100–110)	0.198 a
Diastolic blood pressure (mmhg)	70 (60–75)	65 (60–70)	0.104 a
Triglyceride (mg/dL)	70.5 (56–91)	74 (53.5–102)	0.973 a
Total cholesterol (mg/dL)	159 (142–183)	158 (138.5–184)	0.834 a
HDL cholesterol (mg/dL)	51 (43–58)	51 (45–57)	0.578 a
LDL cholesterol (mg/dL)	91.2 (80.5–111.8)	89 (77.7–111.7)	0.542 a
Glucose (mg/dL)	82 (76.5–87)	82.5 (78–88)	0.280 a
Insulin (miu/L)	9.33 (5.27–14.5)	9.16 (4.98–17.6)	0.877 a
HOMA index	1.78 (1.040–3.038)	1.749 (1.005–3.573)	0.704 a
Visceral adiposity index	1.075 (0.803–1.455)	1.123 (0.792–1.538)	0.879 c
Insulin resistance n (%)	30 (32.6%)	30 (32.6%)	1.000 c

Parametric data were given as mean–standard error and nonparametric data were presented as median (25–75 quartile) and categorical variables were presented as a percentage.

a Mann Whitney U,

b Student–t test,

c Chi-square test,

d Hip circumference and waist-hip ratio of 149 participants were evaluated. BMI: Body mass index, HDL: High-density lipoprotein, LDL: Low-density lipoprotein HOMA: Homeostasis model assessment.

**Table 3:** Comparison of data between control group and acne severity groups.

	Control group (n = 92)	Moderate acne group (n = 50)	Severe acne group (n = 42)	P-value
Age (year)	22 (19–25)	21.5 (20–23)	20 (19–23)	0.241 a
Height (m)	1.65 ± 0.1	1.64 ± 0.01	1.64 ± 0.01	0.266 b
Weight (kg)	59 (54–64.5)	56 (52–61)	57 (52–62)	0.232 a
BMI (kg/m^2^)	21.62 ± 0.26	21.16 ± 0.29	21.17 ± 0.34	0.628 b
Waist circumference (cm)	74 (768.5–79.5)	70 (67–77)	75 (69–80)	0.232 a
Hip circumference (cm)	92.5 (90–97)	92 (87–96)	93 (89–99)	0.493^a^^e^
Waist/hip ratio	0.77 (0.74–0.83)	0.77 (0.75–0.8)	0.79 (0.76–0.84)	0.377 ae
Systolic blood pressure (mmHg)	110 (100–112.5)	105 (100–110)	105 (100–110)	0.424 a
Diastolic blood pressure (mmHg)	70 (60–75)	60 (60–70)	70 (60–70)	0,133^a^
Acne severity score	-	23.3 ± 0.36	33.24 ± 0.26	**<0.05** d
Triglyceride (mg/dL)	70.5 (56–91)	70 (53–107)	75.5 (55–92)	0.996 a
Total cholesterol (mg/dL)	159 (142–183)	161 (145–183)	148 (130–184)	0.370 a
HDL cholesterol (mg/dL)	50.641 ± 1.072	52.920 ± 1.577	50.571 ± 1.393	0.402 b
LDL cholesterol (mg/dL)	91.2 (80–111)	91.7 (79.6–111.4)	88.2 (70–112)	0.616 a
Glucose (mg/dL)	82 (76.5–87)	82 (79–88)	83.5 (77–90)	0.499 a
Insulin (miu/L)	9.33 (14.5–5.27)	8.48 (4.86–14.8)	9.84 (5.1–19.7)	0.666 a
HOMA index	1.78 (1.040–3.038)	1.704 (1.056–3.234)	1.983 (0.982–4.335)	0.670 a
Insulin resistance n (%)	30 (32.6%)	14 (28%)	16 (38.1%)	0.590 c
Visceral adiposity index	1.075 (0.803–1.455)	1.111 (0.779–1.465)	1.134 (0.806–1.586)	0.947 a

Parametric data were given as mean–standard error and nonparametric data were presented as median (25–75 quartile) and categorical variables were presented as a percentage.

a Kruskal-Wallis Test,

b ANOVA,

c The Chi-square,

d 92 people in the patient group were evaluated and the student’s t-test was used to compare the acne severity in 2 groups.

e Hip circumference and waist-hip ratio of 149 participants were evaluated. BMI: body mass index, HDL: high-density lipoprotein, LDL: low-density lipoprotein, HOMA: homeostasis model assessment.

**Table 4:** Correlation analysis between vai, insulin resistance, metabolic syndrome, and some anthropometric parameters in the patient group.

		WC	BMI	HOMA	Insulin	Glucose	TG	HDL	HC	WHR
VAI	r	0.252	0.061	0.298	0.273	0.298	0.856	−0.446	0.260	0.162
	p	**0.015**	0.564	**0.004**	**0.009**	**0.004**	**<0.001**	**<0.001**	**0.019**	0.149
BMI	r	0,500	1000	−0.008	0.003	−0.038	0.097	−0.044	0.582	0.191
	p	**<0.001**	−	0.937	0.978	0.723	0.360	0.674	**<0.001**	0.087
HOMA	r	0.298	−0.008	1000	0.992	0.335	0.263	−0.143	0.079	0.076
	p	**0.004**	0.937	−	**<0.001**	**0.001**	**0.011**	0.175	0.483	0.498
Weight	r	0.579	0.828	0.022	0.023	0.046	0.039	0.004	0.639	0.176
	p	**<0.001**	**<0.001**	0.832	0.830	0.537	0.713	0.973	**<0.001**	0.116

Spearman correlation analysis, VAI: visceral adiposity index, WC: waist circumference, HC: hip circumference, BMI: body mass index, HOMA: homeostasis model assessment, TG: triglyceride, HDL: high-density lipoprotein, WHR: waist/hip ratio.

**Table 5:** Correlation analysis of parameters with acne severity score in the patient group.

	r	p *
Weight (kg)	0.021	0.844
Body mass index (kg/m^2^)	−0.082	0.440
Waist circumference (cm)	0.076	0.474
Hip circumference (cm)	0.012	0.915
Waist/hip ratio	0.104	0.355
Systolic blood pressure (mmhg)	−0.052	0.626
Diastolic blood pressure (mmhg)	0.080	0.449
Triglyceride (mg/dL)	−0.017	0.869
Total cholesterol (mg/dL)	−0.138	0.188
HDL cholesterol (mg/dL)	−0.079	0.456
LDL cholesterol (mg/dL)	−0.110	0.299
Glucose (mg/dL)	0.039	0.713
Insulin (miu/L)	0.057	0.591
HOMA index	0.047	0.659
Visceral adiposity index	−0.001	0.990

* Spearman correlation analysis, HDL: high-density lipoprotein, LDL: low-density lipoprotein, HOMA: homeostasis model assessment.
